# Avelumab in paediatric patients with refractory or relapsed solid tumours: dose-escalation results from an open-label, single-arm, phase 1/2 trial

**DOI:** 10.1007/s00262-022-03159-8

**Published:** 2022-03-09

**Authors:** David M. Loeb, Ji Won Lee, Daniel A. Morgenstern, Yvan Samson, Anne Uyttebroeck, Chuhl Joo Lyu, An Van Damme, Karsten Nysom, Margaret E. Macy, Alexandra P. Zorzi, Julia Xiong, Petra Pollert, Ingrid Joerg, Yulia Vugmeyster, Mary Ruisi, Hyoung Jin Kang

**Affiliations:** 1 Division of Pediatric Hematology, Oncology, and Cellular Therapy, Children’s Hospital at Montefiore, Bronx, NY USA; 2 Department of Pediatrics, Samsung Medical Center, Sungkyunkwan University School of Medicine, Seoul, Republic of Korea; 3 Department of Paediatrics, The Hospital for Sick Children, and University of Toronto, Toronto, ON Canada; 4 Department of Paediatrics, Centre Hospitalier Universitaire Sainte-Justine, University of Montreal, Montreal, QC Canada; 5 Department of Pediatric Hematology and Oncology, University Hospitals Leuven, KU Leuven, Leuven, Belgium; 6 Department of Pediatrics, Yonsei University College of Medicine, Seoul, Republic of Korea; 7 Department of Pediatric Hematology and Oncology, Cliniques Universitaires Saint-Luc, Université Catholique de Louvain, Brussels, Belgium; 8 Department of Paediatrics and Adolescent Medicine, Rigshospitalet, Copenhagen, Denmark; 9 Department of Pediatrics, University of Colorado Anschutz Medical Campus, and Children’s Hospital Colorado, Aurora, CO USA; 10 Children’s Hospital, London Health Sciences Centre, London, ON Canada; 11 EMD Serono Research & Development Institute, Inc., Billerica, MA USA an affiliate of Merck KGaA,; 12 Merck Healthcare KGaA, Darmstadt, Germany; 13 Department of Pediatrics, Seoul National University College of Medicine, Seoul National University Cancer Research Institute, Wide River Institute of Immunology, Seoul National University Children’s Hospital, Seoul, Republic of Korea

**Keywords:** Avelumab, Paediatrics, Phase 1, Immunotherapy, Immune checkpoint inhibitor

## Abstract

**Background:**

We report dose-escalation results from an open-label, phase 1/2 trial evaluating avelumab (anti-PD-L1) in paediatric patients with refractory/relapsed solid tumours.

**Methods:**

In phase 1, patients aged < 18 years with solid (including central nervous system [CNS]) tumours for which standard therapy did not exist or had failed were enrolled in sequential cohorts of 3–6 patients. Patients received avelumab 10 or 20 mg/kg intravenously every 2 weeks. Primary endpoints were dose-limiting toxicities (DLTs) and grade ≥ 3 treatment-emergent adverse events (AEs).

**Results:**

At data cut-off (27 July 2021), 21 patients aged 3–17 years had received avelumab 10 mg/kg (*n* = 6) or 20 mg/kg (*n* = 15). One patient had three events that were classified as a DLT (fatigue with hemiparesis and muscular weakness associated with pseudoprogression; 20 mg/kg cohort). Grade ≥ 3 AEs occurred in five (83%) and 11 (73%) patients in the 10 and 20 mg/kg cohorts, respectively, and were treatment-related in one patient (7%; grade 3 [DLT]) in the 20 mg/kg cohort. Avelumab exposure in paediatric patients receiving 20 mg/kg dosing, but not 10 mg/kg, was comparable or higher compared with approved adult dosing (10 mg/kg or 800 mg flat dose). No objective responses were observed. Four patients with CNS tumours (20 mg/kg cohort) achieved stable disease, which was ongoing in two patients with astrocytoma at cut-off (for 24.7 and 30.3 months).

**Conclusion:**

In paediatric patients with refractory/relapsed solid tumours, avelumab monotherapy showed a safety profile consistent with previous adult studies, but clinical benefits were limited.

**Supplementary Information:**

The online version contains supplementary material available at 10.1007/s00262-022-03159-8.

## Introduction

Treatment of advanced paediatric cancer typically includes cytotoxic chemotherapy; however, patients often develop resistance and have refractory or relapsed disease, resulting in a poor prognosis [1, 2]. Immune checkpoint inhibitors (ICIs) that target the PD-L1/PD-1 interaction have been approved as treatments for various adult cancers. Recently, several early phase trials investigating ICI monotherapy specifically in paediatric cancers have shown acceptable safety profiles but low antitumour activity, except in Hodgkin lymphoma [3–5].

Avelumab, an anti-PD-L1 antibody, has shown clinical activity in various tumours [6–10]. Avelumab is approved in various countries for the treatment of metastatic Merkel cell carcinoma (including patients aged ≥ 12 years in the USA) in addition to platinum-treated urothelial carcinoma (first-line maintenance therapy or second-line therapy) and advanced renal cell carcinoma (first-line treatment in combination with axitinib) [11]. Avelumab was initially approved with 10-mg/kg dosing every 2 weeks (Q2W), but this was subsequently changed to a flat dose of 800 mg in the USA, Europe, and other locations, based on pharmacokinetic (PK) studies [12]. Other ICIs approved specifically for paediatric patients are pembrolizumab (in primary mediastinal large B-cell lymphoma, microsatellite instability—high cancers, tumour mutational burden—high cancers, and Merkel cell carcinoma in the USA, and relapsed/refractory classical Hodgkin lymphoma in the USA and Europe) and nivolumab (alone or combined with ipilimumab in microsatellite instability—high metastatic colorectal cancer in the USA) [13, 14]. Except for Hodgkin lymphoma, ICI approvals in paediatric populations have generally been based on paediatric safety/PK analyses and efficacy findings in adults [13, 14].

We report dose-escalation results from a trial of avelumab monotherapy in paediatric patients with refractory or relapsed solid tumours.

## Methods

### Study design and participants

In phase 1 of this international, open-label, multicentre, single-arm, phase 1/2 trial (registered at clinicaltrials.gov: NCT03451825), eligible patients were aged < 18 years at first dose and had a histologically or cytologically confirmed diagnosis of a solid tumour (including central nervous system [CNS] tumours) or lymphoma that had progressed with standard therapy or for which no standard therapy existed. Other eligibility criteria included Lansky (≤ 16 years) or Karnofsky (> 16 years) performance status ≥ 50; estimated life expectancy > 3 months; adequate haematologic, hepatic, and renal function; availability of recently obtained tumour tissue; negative pregnancy test (in all postmenarcheal females, females aged ≥ 10 years, or per local guidelines); and use of effective contraception (in patients who were considered to be biologically capable of having children and were sexually active). Exclusion criteria included rapidly progressive disease (PD), grade ≥ 3 neuropathy, known congenital immunodeficiency, prior therapy targeting a T-cell coregulatory protein, active autoimmune disease that might deteriorate when receiving an immunostimulatory agent (not including diabetes type 1, vitiligo, psoriasis, or hypothyroid/hyperthyroid disease not requiring immunosuppressive treatment), and serious cardiovascular disease or other severe medical conditions. Use of systemic steroids was tapered before study treatment except for adrenal insufficiency (physiological replacement dose permitted) or acute allergy (≤ 14 days permitted).

The trial was conducted in accordance with the ethics principles of the Declaration of Helsinki and the International Council for Harmonisation Guideline for Good Clinical Practice. The protocol was approved by the institutional review board or independent ethics committee of each centre. All patients or legal representatives of patients provided written informed consent before enrolment.

### Procedures

Sequential cohorts of three to six patients were enrolled. The avelumab starting dose was 10 mg/kg by 1-h intravenous infusion Q2W. Escalation to, but not exceeding, 20 mg/kg intravenously Q2W was planned if exposure was inadequate compared with adult exposures derived from population PK simulations (maximum serum concentration, area under the concentration–time curve [AUC], and trough serum concentration [C_trough_]). To mitigate the potential for infusion-related reactions (IRRs), a known adverse event (AE) with avelumab [15], antihistamine (e.g. diphenhydramine) and paracetamol premedication, dosed per local treatment standards, was mandatory 30 to 60 min before the first four infusions.

AEs were coded using Medical Dictionary for Regulatory Activities (MedDRA) version 22.1 and graded according to the National Cancer Institute Common Terminology Criteria for Adverse Events version 4.03. Dose-limiting toxicity (DLT) was defined as any of the following events occurring during the DLT observation period (first two cycles of treatment [28 days]) if considered related to avelumab: grade 4 neutropenia (> 7 days), thrombocytopenia (> 7 days), or anaemia; grade ≥ 3 neutropenic infection or thrombocytopenia with bleeding; or specified grade ≥ 3 nonhaematologic toxicities excluding those that resolved and/or were without clinical correlate. Inability to complete two or more avelumab infusions during the DLT period due to treatment-related toxicity was also classified as a DLT. All safety data were reviewed by the Safety Monitoring Committee (SMC) for potential DLTs at predefined intervals. The maximum tolerated dose (MTD) was defined as the highest dose level at which < 33% of evaluable patients experienced a DLT, provided that a higher dose level was tested and had an associated DLT rate ≥ 33%. Immune-related AEs were evaluated using a customised list of MedDRA terms and by investigator assessment using a predefined medical algorithm. IRRs were identified using prespecified lists of MedDRA terms in association with time of onset and resolution.

Patients received avelumab until confirmed PD per immune-related Response Evaluation Criteria in Solid Tumours (irRECIST), death, unacceptable toxicity, or withdrawal of consent. Treatment could continue after confirmed PD if the patient had no new or worsening symptoms, was tolerating avelumab, had stable performance status, and treatment would not delay preventive intervention for serious complications of PD. Tumours were assessed radiologically at baseline, every 8 weeks for 24 weeks, then every 12 weeks thereafter. Objective tumour response was evaluated by investigators per RECIST version 1.1. For some analyses, patients were assigned to subgroups of CNS tumours, sarcomas, and gastrointestinal (GI) tumours.

Blood samples for PK analysis were collected during treatment cycles 1, 2, 3, 5, 7, 8, 13, and every 6 cycles thereafter. Serum avelumab concentrations were analysed by immunoassay. PK parameters were calculated by noncompartmental analysis.

PD-L1 expression was assessed in baseline tumour tissue using the PD-L1 73–10 immunohistochemistry assay (Dako, Carpinteria, California, USA). PD-L1+ status was defined as PD-L1 expression on tumour cells at any intensity with cut-offs of ≥ 1%, ≥ 5%, ≥ 25%, ≥ 50%, or ≥ 80%.

### Outcomes

Primary endpoints in phase 1 were DLTs in the DLT observation period, to determine the recommended phase 2 dose, and grade ≥ 3 treatment-emergent AEs. Secondary endpoints included confirmed best overall response and progression-free survival (PFS) per RECIST 1.1 by investigator assessment; overall survival (OS); safety; and single/multiple-dose PK profiles.

### Statistical analysis

Efficacy and safety were analysed in all patients who received at least one avelumab dose. DLTs were evaluated in all patients who received all assigned trial treatment administrations in the DLT evaluation period or who stopped treatment because of DLTs in this period. Planned enrolment was 12 to 36 patients in phase 1 using the modified toxicity probability interval approach [16]. At least 12 DLT-evaluable patients, treated at a dose level confirmed to be safe, were required for the primary analysis. Two-sided 95% CIs for objective response rates were calculated using the Clopper–Pearson method. Time-to-event endpoints were estimated using the Kaplan–Meier method, and corresponding two-sided CIs for medians were calculated using the Brookmeyer–Crowley method.

## Results

### Patients

Twenty-one patients with various advanced solid tumours were enrolled. Most patients (71%) were Asian. Median age was 12.0 years (range 3–17), and median weight was 37.3 kg (range 13.4–78.7). Patients received avelumab 10 mg/kg (*n* = 6) or 20 mg/kg (*n* = 15) Q2W (Table 1). Tumour subgroups were CNS in eight (all 20 mg/kg cohort), sarcoma in 12 (10 mg/kg [*n* = 5] and 20 mg/kg [*n* = 7] cohorts), and GI in one (colon cancer; 10 mg/kg cohort). No patients with lymphoma were enrolled. All patients had received prior therapy; nine patients (43%) had received four or more prior lines of therapy (Table 1).

**Table 1:** Baseline characteristics

	Avelumab 10 mg/kg (*n* = 6)	Avelumab 20 mg/kg (*n* = 15)	All patients (*N* = 21)
Sex, *n* (%)			
Male	4 (67)	7 (47)	11 (52)
Female	2 (33)	8 (53)	10 (48)
Race, *n* (%)			
Asian	6 (100)	9 (60)	15 (71)
White	0	4 (27)	4 (19)
Data not collected	0	2 (13)	2 (10)
Geographical region, *n* (%)			
North America	0	5 (33)	5 (24)
Western Europe	0	1 (7)	1 (5)
Asia	6 (100)	9 (60)	15 (71)
Median age, years (range)	12.5 (8–15)	12.0 (3–17)	12.0 (3–17)
Age category, *n* (%)			
1–12 years	3 (50)	8 (53)	11 (52)
> 12 years	3 (50)	7 (47)	10 (48)
Median weight, kg (range)	34.6 (18.5–65.6)	37.3 (13.4–78.7)	37.3 (13.4–78.7)
Primary tumour category, *n* (%)			
Central nervous system	0	8 (53)	8 (38)
Soft tissue/bone sarcoma	5 (83)	7 (47)	12 (57)
Gastrointestinal (colon cancer)	1 (17)	0	1 (5)
Median time since initial cancer diagnosis, months (range)	21.1 (4.5–60.2)	24.1 (4.3–168.0)	22.2 (4.3–168.0)
Median time since diagnosis of metastatic disease, months (range)	14.5 (2.6–29.7)	6.2 (0.3–53.5)	10.3 (0.3–53.5)
Disease stage at study entry, *n* (%)			
I	0	0	0
II	0	1 (7)	1 (5)
III	1 (17)	0	1 (5)
IV	5 (83)	8 (53)	13 (62)
Missing	0	6 (40)	6 (29)
Prior anticancer surgery, *n* (%)	6 (100)	15 (100)	21 (100)
Prior anticancer radiotherapy, *n* (%)	3 (50)	7 (47)	10 (48)
No. of prior anticancer drug regimens, *n* (%)			
0	0	0	0
1	1 (17)	2 (13)	3 (14)
2	0	7 (47)	7 (33)
3	1 (17)	1 (7)	2 (10)
≥ 4	4 (67)	5 (33)	9 (43)
Best overall response to prior anticancer therapy, *n* (%)^a^			
Complete response	0	1 (7)	1 (5)
Partial response	0	2 (13)	2 (10)
Stable disease	2 (33)	0	2 (10)
Progressive disease	3 (50)	8 (53)	11 (52)
Not evaluable	1 (17)	0	1 (5)
Unknown	0	4 (27)	4 (19)

^a^If a patient had received more than one prior anticancer therapy, best overall response to last therapy received is reported

**Fig. 1:**
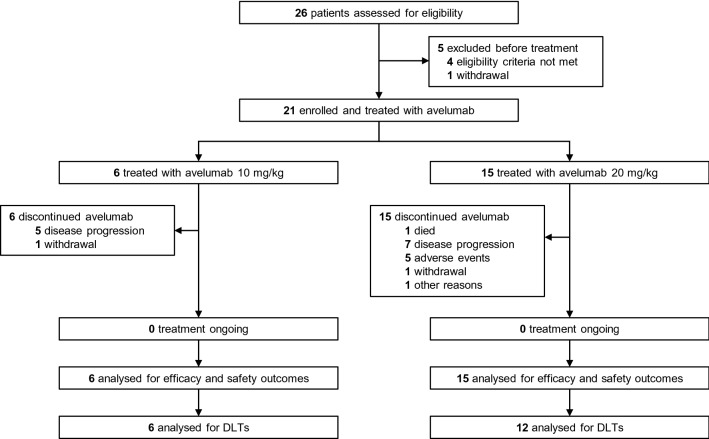
Trial profile. DLT, dose-limiting toxicity

Median duration of treatment was 8.2 weeks (range 6.1–15.9) in the 10 mg/kg cohort and 11.9 weeks (range 2.0–134.1) in the 20 mg/kg cohort, and median follow-up was 18.8 weeks (range 6.4–62.3) and 30.1 weeks (range 3.6–139.0), respectively. At data cut-off (27 July 2021), no patient remained on treatment (Fig. 1). The most common reason for discontinuation was PD (10 mg/kg, *n* = 5 [83%]; 20 mg/kg, *n* = 7 [47%]) (Fig. 1).

### Safety

One patient in the 20 mg/kg cohort was not included in the DLT analysis because they received only one dose of avelumab owing to an AE. Of the remaining 20 patients (10 mg/kg, *n* = 6; 20 mg/kg, *n* = 14), 18 completed the DLT-evaluable period, whereas two patients stopped treatment after receiving two doses of avelumab due to PD, who, therefore, were nonevaluable for DLTs. One patient (8%) in the 20 mg/kg cohort with a high-grade glioma experienced three concurrent events (fatigue with hemiparesis and muscular weakness associated with pseudoprogression; all grade 3) that were assessed as a DLT by the SMC. The MTD was not reached. During the DLT evaluation period, treatment-emergent AEs of any grade or causality occurred in all six patients (100%) in the 10 mg/kg cohort and 11 of 12 patients (92%) in the 20 mg/kg cohort, including grade ≥ 3 AEs in one patient (17%) and two patients (17%), respectively.

In the full patient group, AEs of any grade occurred in all 21 patients, including grade ≥ 3 AEs in five patients (83%) in the 10 mg/kg cohort and 11 patients (73%) in the 20 mg/kg cohort (Table 2). The most common grade ≥ 3 AEs (≥ 30%) were abdominal pain (*n* = 2 [33%]) in the 10 mg/kg cohort and disease progression (*n* = 5 [33%]) in the 20 mg/kg cohort. In the 10 and 20 mg/kg cohorts, serious AEs of any grade occurred in four patients (67%) and 12 patients (80%), respectively. AEs led to discontinuation in patients in the 20 mg/kg cohort only (*n* = 8 [53%]), including disease progression (*n* = 5 [33%]), and thrombocytopenia (*n* = 1 [7%]), malignant pleural effusion (*n* = 1 [7%]), and intracranial pressure increased (*n* = 1 [7%]), all three of which were related to disease progression. AEs resulted in death in one patient (17%) in the 10 mg/kg cohort and three patients (20%) in the 20 mg/kg cohort, all due to disease progression. Treatment-related AEs (TRAEs) of any grade occurred in three patients (50%) in the 10 mg/kg cohort and 10 patients (67%) in the 20 mg/kg cohort (Supplementary Table 1). The most common TRAEs (≥ 20%) in the 20 mg/kg cohort were fatigue (*n* = 4 [27%]), nausea (*n* = 3 [20%]), and chills (*n* = 3 [20%]); no TRAE occurred in more than one patient in the 10 mg/kg cohort. Grade 3 TRAEs occurred in one patient (7%) in the 20 mg/kg cohort (fatigue, hemiparesis, muscle weakness, and tumour pseudoprogression; patient with DLT described above). No grade 4 or 5 TRAEs occurred, and none led to discontinuation. An immune-related AE occurred in one patient (7%) in the 20 mg/kg cohort (grade 2 hypothyroidism). Grade 1/2 IRRs occurred in two patients (33%) in the 10 mg/kg cohort and seven patients (47%) in the 20 mg/kg cohort; no grade ≥ 3 IRRs occurred.

**Table 2:** Treatment-emergent adverse events

	Avelumab 10 mg/kg (*n* = 6)		Avelumab 20 mg/kg (*n* = 15)	
	Any grade	Grade ≥ 3	Any grade	Grade ≥ 3
Any AE, *n* (%)	6 (100)	5 (83)	15 (100)	11 (73)
Pyrexia	4 (67)	0	10 (67)	0
Anaemia	2 (33)	1 (17)	5 (33)	1 (7)
Abdominal pain	2 (33)	2 (33)	3 (20)	0
Disease progression	1 (17)	1 (17)	5 (33)	5 (33)
Dyspnoea	1 (17)	1 (17)	3 (20)	1 (7)
Hyponatraemia	1 (17)	1 (17)	2 (13)	2 (13)
Vomiting	1 (17)	0	6 (40)	0
Back pain	1 (17)	0	4 (27)	1 (7)
Constipation	1 (17)	0	5 (33)	0
Hypoalbuminemia	1 (17)	0	4 (27)	0
Arthralgia	1 (17)	0	3 (20)	1 (7)
Chills	1 (17)	0	3 (20)	0
Hypotension	1 (17)	0	3 (20)	0
Fatigue	0	0	6 (40)	1 (7)
Nausea	0	0	6 (40)	1 (7)
Headache	0	0	5 (33)	1 (7)
Pain in extremity	0	0	4 (27)	0
Hypophagia	0	0	3 (20)	2 (13)
Nasopharyngitis	0	0	3 (20)	0
Procedural pain	0	0	3 (20)	0
Pruritus	0	0	3 (20)	0
Hypertension	0	0	2 (13)	2 (13)

AEs of any grade occurring in three or more patients or grade ≥ 3 in two or more patients in either cohort are shown

AE, treatment-emergent adverse event

### PK

In PK analyses (*N* = 21; data cut-off, 21 October 2019), the median and geometric mean of the AUC and C_trough_ for cycle 1 in the 10 mg/kg cohort appeared lower vs approved adult dosing, particularly in patients with a body weight of < 40 kg (Table 3). The median and geometric mean of the AUC and C_trough_ for cycle 1 in the 20 mg/kg cohort were similar or higher vs adult values with approved dosing, irrespective of body weight. No clear association was observed between age and exposure in either dose cohort. Additionally, the PK profile in the patient with DLT was similar to other patients in the same dose cohort (20 mg/kg) and adults treated with approved dosing.

**Table 3:** PK summary following first infusion of cycle 1 of avelumab in paediatric patients, with adult data shown for comparison

Dose cohort, body weight category	Patients, *n*	C_max_, µg/mL		AUC_0-336_, µg·h/mL		C_trough_, µg/mL	
		Geometric mean	Geometric CV, %	Geometric mean	Geometric CV, %	Geometric mean	Geometric CV, %
800 mg, adults	10,000	256	25.6	24,486	27.7	17.2	68
10 mg/kg, all patients	6	190	34.5	18,800	29.2	11.2	44.9
10 mg/kg, < 40 kg	4	157	16.2	16,000	19.1	8.8	23.6
10 mg/kg, ≥ 40 kg	2	281	16.9	25,700	7.3	18.3	20
20 mg/kg, all patients	15 (14 for C_trough_)	384	27.3	42,800	22.1	34.8	77.8
20 mg/kg, < 40 kg	10	338	20.4	41,400	21.8	39.4	70.1
20 mg/kg, ≥ 40 kg	5 (4 for C_trough_)	496	19.4	45,900	23.7	25.5	97.5

AUC_0-336_, area under the concentration–time curve from time 0 to 336 h; C_max_, maximum serum concentration; C_trough_, trough serum concentration; CV, coefficient of variation; and PK, pharmacokinetic

Data cut-off, 21 October 2019

### Efficacy

**Fig. 2:**
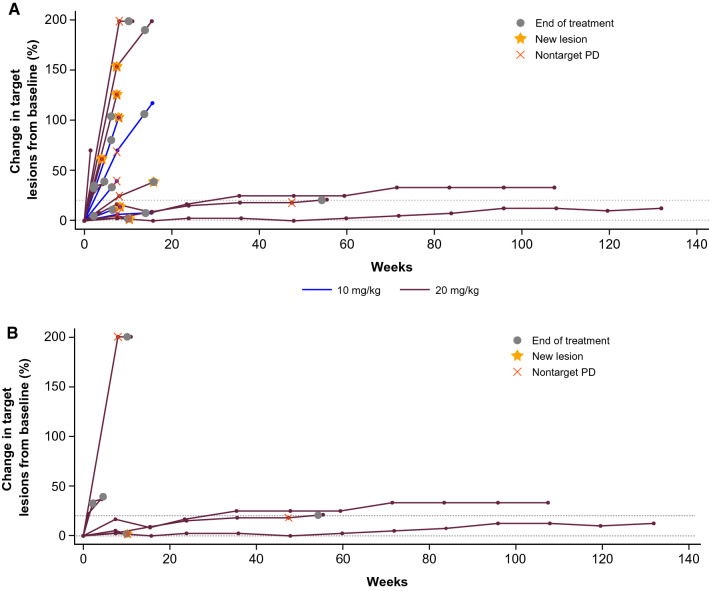
Change in target lesions per RECIST 1.1 from baseline over time in evaluable patients (those with baseline and postbaseline data): **A** all evaluable patients (*n* = 18); **B** patients with central nervous system tumours (*n* = 7). Increases greater than 200% are shown as 200%. PD, progressive disease; RECIST 1.1, Response Evaluation Criteria in Solid Tumours version 1.1

No objective responses were observed (Supplementary Tables 2 and 3). No patient had a reduction in the sum of target lesions (Fig. 2; Supplementary Fig. 1), and no trend in type of progression (i.e. target vs nontarget vs new lesion) was observed (Supplementary Table 4). Four patients in the 20 mg/kg cohort had stable disease (SD). The disease control rate (proportion with confirmed response or SD) was 0% (95% CI, 0–46) in the 10 mg/kg cohort and 27% (95% CI, 8–55) in the 20 mg/kg cohort. All four patients who had SD had a CNS tumour: astrocytoma of the spinal cord, pilocytic astrocytoma, pilomyxoid astrocytoma (all low grade), and H3 K27M-mutant diffuse midline glioma. Duration of SD ranged from 2.4 to 30.3 months and was ongoing at last assessment (data cut-off, 27 July 2021) in two patients with astrocytoma after 30.3 and 24.7 months (Supplementary Fig. 2A). Prestudy target lesion data suggested that these tumours were growing slowly prior to study entry (Supplementary Fig. 2B). Prior systemic treatment or radiotherapy and site of primary tumour were not associated with clinical benefit from avelumab (Supplementary Table 5).

Median PFS was 7.5 weeks (95% CI, 6.6–not estimable [NE]) in the 10 mg/kg cohort and 7.7 weeks (95% CI, 2.3–10.3) in the 20 mg/kg cohort (Supplementary Fig. 3A); median OS was 4.4 months (95% CI, 1.5–NE) and 7.0 months (95% CI, 1.6–10.8), respectively (Supplementary Fig. 3B).

### Biomarker analyses

**Fig. 3:**
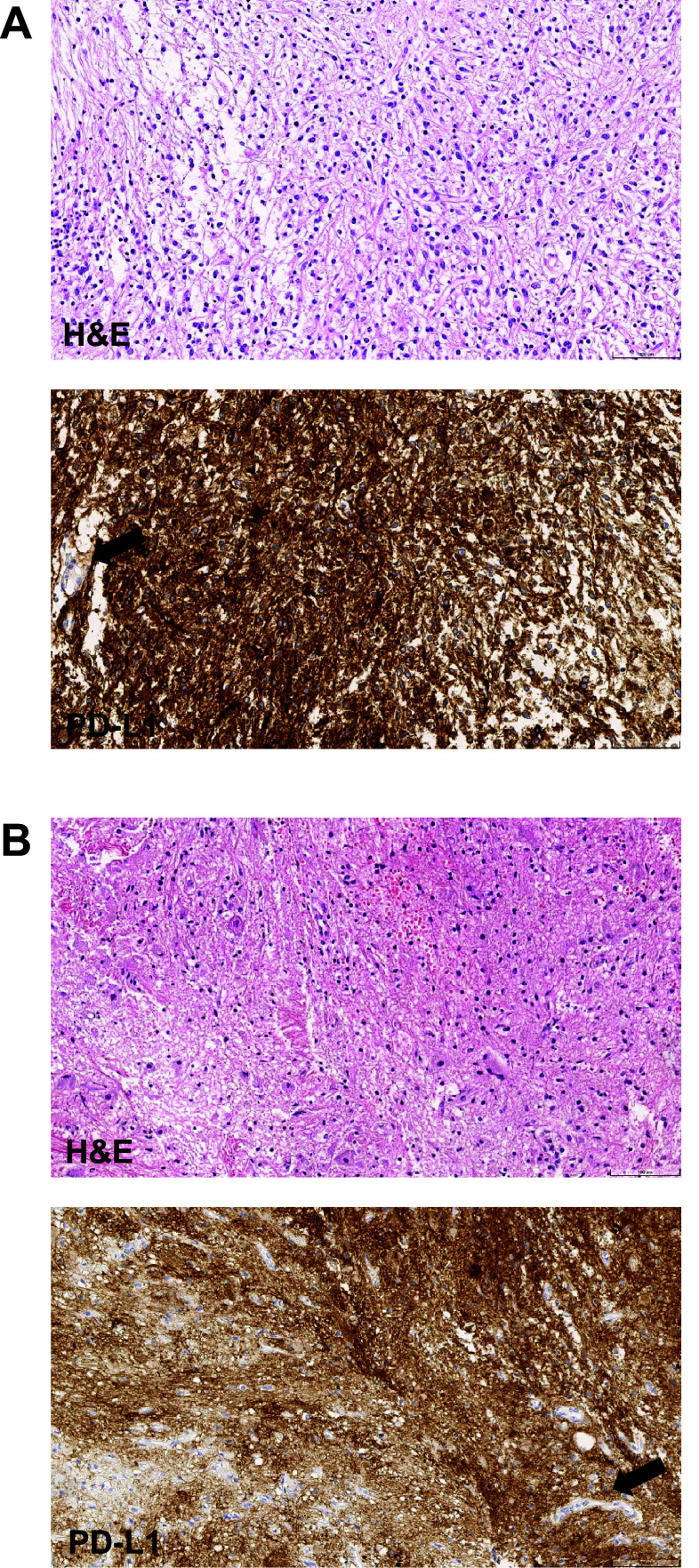
Histological images of H&E and PD-L1 (73–10) staining of tumour samples for the two patients with astrocytoma who had prolonged SD (> 24 months) with avelumab. Both patients had tumours with ≥ 80% of tumour cells having membrane staining positive for PD-L1 expression. Patient A (female aged 9 years) had a pilocytic astrocytoma (WHO grade I). The patient presented in 2018 with a mass at the cerebellopontine angle and upper cervical spine showing cystic and contrast-enhancing solid portions via MRI scan. Histopathology showed an astrocytic tumour with increased cellularity, mild pleomorphism, low mitotic activity (2/10 high-power fields), and absent necrosis. Immunophenotype was positive for glial fibrillary acidic protein and strong PD-L1 expression in tumour cells but not tumour vessels (arrow). The tumour was BRAF^V600E^ mutation-positive, but no PTEN deletion or MGMT promoter methylation was present. The patient underwent surgery in April 2018 with residual tumour and received vincristine + carboplatin from May to July 2018 (best overall response of PD) followed by thioguanine + procarbazine + lomustine in August 2018 (best overall response unknown); no radiation was administered. The patient received avelumab treatment from October 2018 until April 2021, and tumour size changed over time from 40 to 45 mm. Lansky performance status improved from 50% at study entry to 70% with avelumab treatment, and the patient discontinued from the study to receive subsequent anticancer therapy (surgery). Patient B (male aged 3 years) had an astrocytoma of spinal cord (WHO grade II; NF1-associated). The patient presented in 2018 with a contrast-enhancing intramedullary mass at the upper thoracic spinal cord (MRI). Microscopy showed a tumour with increased cellularity, mild pleomorphism, low mitotic activity (1/10 high-power field), absent microvascular proliferation, and absent necrosis. Immunophenotype was positive for glial fibrillary acidic protein and synaptophysin, and strong PD-L1 expression was seen in tumour cells but not tumour vessels (arrow). The tumour had an NF1 mutation (p.Gln1577*, c.4729C > T), but no mutations of BRAF (V600E), IDH1, TP53, or PTEN were present. In 2021, the tumour was classified as ganglioglioma, WHO grade I. The patient had surgery with residual tumour in July 2018 and received vincristine + carboplatin from August to November 2018 (best overall response of PD), with no radiation. The patient received avelumab treatment from December 2018 until February 2021, and tumour size changed over time from 12 to 16 mm (not classified as PD according to RECIST 1.1 because the tumour size did not increase by ≥ 5 mm), with Lansky performance status stable at 90%. The patient discontinued the study to receive subsequent anticancer therapy (surgery). H&E, haematoxylin and eosin; MRI, magnetic resonance imaging; PD, progressive disease; RECIST, Response Evaluation Criteria in Solid Tumours; SD, stable disease; and WHO, World Health Organization

A total of 15 patients were evaluable for PD-L1 expression (Supplementary Table 6). Using a ≥ 1% cut-off to define PD-L1+ status, five patients (33%) had PD-L1+ tumours and 10 (67%) had PD-L1− tumours. Notably, the two patients with astrocytoma who had prolonged and ongoing SD with avelumab treatment had tumours with high PD-L1+ expression at baseline (≥ 80% of tumour cells; Fig. 3). The other three patients with PD-L1+ tumours all had PD as their best overall response with avelumab.

## Discussion

Avelumab monotherapy had an acceptable safety profile in paediatric patients at both dose levels investigated, 10 mg/kg and 20 mg/kg Q2W, with a low incidence of grade ≥ 3 TRAEs and no grade ≥ 3 immune-related AEs. One patient treated with 20 mg/kg had three concurrent events that were assessed as a DLT but were likely associated with tumour pseudoprogression, a known phenomenon with ICI treatment [17] that may not be dose dependent. The MTD was not reached, which has been a common finding in ICI trials and reflects the challenges of dose evaluation using study designs adopted initially for cytotoxic agents. Grade ≥ 3 AEs occurred in 83% in the 10 mg/kg cohort and 73% in the 20 mg/kg cohort, but grade ≥ 3 AEs were considered treatment-related only in one patient (DLT; 20 mg/kg cohort), and no grade 4/5 TRAEs occurred. All AEs that led to discontinuation were associated with disease progression. Grade 1/2 IRRs occurred in 33% in the 10 mg/kg cohort and 47% in the 20 mg/kg cohort, and no grade ≥ 3 IRRs occurred. These rates appear higher than those seen in studies of avelumab in adults, although this may be due to the small sample size in the paediatric study [15]. The frequency of IRRs in this study was also higher than reported for other ICIs in trials in paediatric patients, although it should be noted that trials of other ICIs used narrower definitions for IRR [3, 4]. No new safety signals were observed in paediatric patients, and the frequency and severity of AEs were generally consistent with adult studies [15].

PK analysis showed that paediatric dosing with 10 mg/kg resulted in lower exposure vs adults receiving approved dosing (10 mg/kg or 800 mg Q2W), particularly in patients weighing < 40 kg (i.e. those receiving the lowest dose). However, 20 mg/kg Q2W dosing achieved or exceeded exposures in adults, irrespective of body weight. PK analyses from this study, in addition to subsequent modelling and simulation approaches, have been used to select the recommended dose for future avelumab studies in paediatric patients of 15 mg/kg Q2W for patients < 12 years or < 40 kg and the adult dose of 800 mg Q2W for paediatric patients ≥ 12 years and ≥ 40 kg [18].

Antitumour activity of avelumab monotherapy was limited in relapsed or refractory paediatric solid tumours, consistent with other recent studies of ICIs in similar populations [3–5]. Four patients with CNS tumours achieved SD on study, including two patients with low-grade astrocytoma who had ongoing SD lasting > 24 months; however, prestudy tumour assessments suggested that these tumours were growing slowly. The lack of objective responses reported with several ICIs may be due to differences in tumour biology between paediatric and adult cancers, including a lower mutational burden in most paediatric tumours [19], and differences in immune responses between adults and younger patients [20]. Additionally, the enrolled population had a high proportion of patients who were Asian, which may have introduced bias. Despite recruitment efforts, no patients were enrolled with lymphoma, a malignancy that often responds to ICI monotherapy [3–5]. Limited data are available on paediatric patients with CNS tumours treated with other ICIs because trials generally exclude these patients [4, 5]. In KEYNOTE-051, pembrolizumab showed some benefit in paediatric patients with various PD-L1+ solid tumours, including partial response in a patient with a malignant ganglioglioma, and tumour shrinkage (< 30% decrease) in patients with high-grade glioma, glioblastoma, ependymoma, and ganglioneuroblastoma among other tumours [3]. In this study, two of the three patients with astrocytoma who had prolonged SD with avelumab had high PD-L1+ tumours (≥ 80%); the other three patients with PD-L1+ tumours had PD as best response to avelumab.

This study was part of a paediatric investigation plan approved by the European Medicines Agency in 2017, which was originally for the treatment of solid tumours and was subsequently modified to include lymphomas and CNS tumours [21]. The trial was initiated, and planned as a phase 1/2 study, before the updated overall paediatric strategy for ICIs was agreed upon by ACCELERATE and the European Medicines Agency at the Paediatric Strategy Forum in September 2018, which recommended a focus on combination studies because of the limited activity seen in several studies with ICI monotherapy [22]. Subsequently, it was decided not to proceed with phase 2 after the completion of phase 1 of this trial. A future study will investigate the combination of avelumab plus lenvatinib (a receptor tyrosine kinase inhibitor) in paediatric patients with CNS tumours (NCT05081180). This planned trial is supported by the disease stabilizations observed both in our trial and in a retrospective study of ICIs [23]. Additionally, in a cohort of 31 adults with previously treated glioblastoma multiforme who received lenvatinib in combination with pembrolizumab within a phase 2 trial, an objective response rate of 16% (per Response Assessment in Neuro-Oncology criteria) and disease control rate of 58% were reported [24], supporting the evaluation of lenvatinib and avelumab combination therapy in paediatric patients with CNS tumours.

In conclusion, the tolerability seen with avelumab monotherapy in paediatric patients with previously treated solid tumours, including those with CNS tumours, supports further studies of avelumab-based combination therapy in these tumours.

### Supplementary Information

Below is the link to the electronic supplementary material.

Supplementary file1 (DOCX 522 kb)

## Data Availability

Any requests for data by qualified scientific and medical researchers for legitimate research purposes will be subject to Merck’s Data Sharing Policy. All requests should be submitted in writing to Merck’s data sharing portal (https://www.merckgroup.com/en/research/our-approach-to-research-and-development/healthcare/clinical-trials/commitment-responsible-data-sharing.html). When Merck has a co-research, co-development, or co-marketing or co-promotion agreement, or when the product has been out-licensed, the responsibility for disclosure might be dependent on the agreement between parties. Under these circumstances, Merck will endeavour to gain agreement to share data in response to requests.

## Abbreviations

AE: Adverse event
AUC: Area under the concentration–time curve
CNS: Central nervous system
C_trough_: Trough serum concentration
DLT: Dose-limiting toxicity
GI: Gastrointestinal
H&E: Haematoxylin and eosin
ICI: Immune checkpoint inhibitor
IRR: Infusion-related reaction
irRECIST: Immune-related Response Evaluation Criteria in Solid Tumours
MedDRA: Medical Dictionary for Regulatory Activities
MRI: Magnetic resonance imaging
MTD: Maximum tolerated dose
NE: Not estimable
OS: Overall survival
PD: Progressive disease
PFS: Progression-free survival
PK: Pharmacokinetic
Q2W: Every 2 weeks
RECIST: Response Evaluation Criteria in Solid Tumours
SD: Stable disease
SMC: Safety Monitoring Committee
TRAE: Treatment-related adverse event
WHO: World Health Organization
