# Supplementary material for: Avelumab in paediatric patients with refractory or relapsed solid tumours: dose-escalation results from an open-label, single-arm, phase 1/2 trial
Source: Cancer Immunol Immunother. 2022 Mar 9;71(10):2485–95. doi: 10.1007/s00262-022-03159-8 (PMC9463244; doi:10.1007/s00262-022-03159-8)
Supplement: Supplementary file 1 — Supplementary file1 (DOCX 522 kb) [file 262_2022_3159_MOESM1_ESM.docx]

**Appendix A.** **Supplementary data**

**Supplementary Table 1.** Treatment-related adverse events.

|  | **Avelumab 10 mg/kg (n=6)** | | **Avelumab 20 mg/kg (n=15)** | |
| --- | --- | --- | --- | --- |
|  | **Any grade** | **Grade ≥3** | **Any grade** | **Grade ≥3** |
| Any TRAE, n (%) | 3 (50) | 0 | 10 (67) | 1 (7) |
| Chills | 1 (17) | 0 | 3 (20) | 0 |
| Pyrexia | 1 (17) | 0 | 1 (7) | 0 |
| Asthenia | 1 (17) | 0 | 0 | 0 |
| Lymphoedema | 1 (17) | 0 | 0 | 0 |
| Fatigue | 0 | 0 | 4 (27) | 1 (7) |
| Nausea | 0 | 0 | 3 (20) | 0 |
| Pruritus | 0 | 0 | 2 (13) | 0 |
| Vomiting | 0 | 0 | 2 (13) | 0 |
| Hemiparesis | 0 | 0 | 1 (7) | 1 (7) |
| Muscular weakness | 0 | 0 | 1 (7) | 1 (7) |
| Tumour pseudoprogression | 0 | 0 | 1 (7) | 1 (7) |
| Arthralgia | 0 | 0 | 1 (7) | 0 |
| Cystitis noninfective | 0 | 0 | 1 (7) | 0 |
| Headache | 0 | 0 | 1 (7) | 0 |
| Hypotension | 0 | 0 | 1 (7) | 0 |
| Hypothyroidism | 0 | 0 | 1 (7) | 0 |
| Influenza-like illness | 0 | 0 | 1 (7) | 0 |
| Infusion-related reaction | 0 | 0 | 1 (7) | 0 |
| Lethargy | 0 | 0 | 1 (7) | 0 |
| Memory impairment | 0 | 0 | 1 (7) | 0 |
| Nephritis | 0 | 0 | 1 (7) | 0 |
| Paraesthesia | 0 | 0 | 1 (7) | 0 |
| Pyuria | 0 | 0 | 1 (7) | 0 |
| Rash maculopapular | 0 | 0 | 1 (7) | 0 |
| Sinus tachycardia | 0 | 0 | 1 (7) | 0 |
| Urticaria | 0 | 0 | 1 (7) | 0 |

TRAE, treatment-related adverse event.

**Supplementary Table 2.** Confirmed best overall response.

|  | **Avelumab 10 mg/kg**  **(n=6)** | **Avelumab 20 mg/kg**  **(n=15)** |
| --- | --- | --- |
| Best overall response by investigator, n (%)  Confirmed response  Partial response  Stable disease  Progressive disease  Not evaluable | 0  0  0  5 (83)  1 (17) | 0  0  4 (27)  9 (60)  2 (13) |
| Objective response rate, % (95% CI) | 0 (0–46) | 0 (0–22) |
| Disease control rate, % (95% CI) | 0 (0–46) | 27 (8–55) |

**Supplementary Table 3.** Baseline characteristics and outcomes in the (A) 10 mg/kg and (B) 20 mg/kg cohorts.

**A**

| **Assigned patient number** | **Age, years** | **Sex** | **Race** | **Weight, kg** | **Tumour type** | **Category** | **Stage** | **PS** | **BOR** | **Vital status at data  cutoff^a^** |
| --- | --- | --- | --- | --- | --- | --- | --- | --- | --- | --- |
| 1 | 15 | Male | Asian | 65.6 | Colon cancer, sigmoid | GI | IV | L, 100% | PD | Dead |
| 2 | 15 | Male | Asian | 30.7 | Desmoplastic small round cell tumour | Soft tissue/bone sarcoma | IV | L, 50% | PD | Dead |
| 3 | 11 | Female | Asian | 38.5 | Osteogenic sarcoma | Soft tissue/bone sarcoma | IV | L, 50% | PD | Dead |
| 4 | 14 | Male | Asian | 60.8 | Rhabdomyosarcoma | Soft tissue/bone sarcoma | III | L, 80% | PD | Dead |
| 5 | 8 | Female | Asian | 18.5 | Rhabdomyosarcoma | Soft tissue/bone sarcoma | IV | L, 80% | NE^b^ | Dead |
| 6 | 9 | Male | Asian | 26.4 | Rhabdomyosarcoma | Soft tissue/bone sarcoma | IV | L, 90% | PD | Dead |

**B**

| **Assigned patient number** | **Age, years** | **Sex** | **Race** | **Weight, kg** | **Tumour type** | **Category** | **Stage** | **PS** | **BOR** | **Vital status at data  cutoff^a^** |
| --- | --- | --- | --- | --- | --- | --- | --- | --- | --- | --- |
| 7 | 17 | Male | White | 78.7 | *BCOR-CCNB3* sarcoma | Soft tissue/bone sarcoma | IVA | K, 90% | PD | Dead |
| 8 | 16 | Female | White | 66.8 | Osteosarcoma | Soft tissue/bone sarcoma | IVB | L, 80% | NE^c^ | Dead |
| 9 | 6 | Female | White | 22.4 | Atypical teratoid/rhabdoid tumour | CNS | Missing | L, 100% | PD | Dead |
| 10 | 16 | Female | White | 49.5 | Undifferentiated sarcoma | Soft tissue/bone sarcoma | IV | L, 100% | PD | Dead |
| 11 | 13 | Female | Not collected | 60.9 | Glioblastoma | CNS | IV | L, 100% | PD | Dead |
| 12 | 14 | Female | Not collected | 37.3 | High-grade glioma; WHO grade IV | CNS | IV | L, 80% | PD | Dead |
| 13 | 11 | Male | Asian | 35.3 | Ewing sarcoma | Soft tissue/bone sarcoma | Missing | L, 60% | PD | Dead |
| 14 | 9 | Female | Asian | 36.7 | Pilocytic astrocytoma; *BRAF* V600E mutation positive | CNS | Missing | L, 50% | SD | Alive |
| 15 | 3 | Male | Asian | 16.6 | Astrocytoma of spinal cord (WHO grade II); *BRAF* V600E mutation negative | CNS | Missing | L, 90% | SD | Alive |
| 16 | 13 | Male | Asian | 39.5 | Rhabdomyosarcoma | Soft tissue/bone sarcoma | IV | L, 60% | NE^d^ | Dead |
| 17 | 16 | Female | Asian | 43.5 | Malignant peripheral nerve sheath tumour/ schwannoma/acoustic neuroma | Soft tissue/bone sarcoma | II | L, 80% | PD | Dead |
| 18 | 9 | Male | Asian | 15.3 | Rhabdomyosarcoma | Soft tissue/bone sarcoma | IV | L, 60% | PD | Dead |
| 19 | 3 | Male | Asian | 16.7 | Anaplastic ependymoma | CNS | IV | L, 80% | PD | Dead |
| 20 | 3 | Female | Asian | 13.4 | Pilomyxoid astrocytoma; *BRAF* V600E mutation negative | CNS | Missing | L, 90% | SD | Alive |
| 21 | 12 | Male | Asian | 37.3 | Diffuse midline glioma (primary site, thalamus); WHO grade IV; H3 K27M mutation | CNS | Missing | L, 70% | SD | Dead |

BOR, best overall response; CNS, central nervous system; GI, gastrointestinal; K, Karnofsky; L, Lansky; NE, not evaluable; PD, progressive disease; PS, performance score; SD, stable disease; WHO, World Health Organization.

^a^ Data cutoff: 27 July 2021.

^b^ No postbaseline assessments because of death due to clinical progression.

^c^ No postbaseline assessments because of death.

^d^ Patient had an early unscheduled tumour assessment and died 2 weeks later due to clinical progression; no radiological progression was recorded.

**Supplementary Table 4.** Details of lesion progression in evaluable patients.

| **Assigned patient number** | **Tumour category** | **Target lesion progression** | **Nontarget lesion progression** | **New lesion** |
| --- | --- | --- | --- | --- |
| 1 | GI (colon cancer) |  | X | X |
| 2 | Soft tissue/bone sarcoma |  | X |  |
| 3 | Soft tissue/bone sarcoma | X | X |  |
| 4 | Soft tissue/bone sarcoma | X | X |  |
| 6 | Soft tissue/bone sarcoma | X | X | X |
| 7 | Soft tissue/bone sarcoma | X | X | X |
| 9 | CNS (ATRT) | NA^a^ | NA^a^ | X |
| 10 | Soft tissue/bone sarcoma | X |  |  |
| 11 | CNS (glioblastoma) | X |  |  |
| 12 | CNS (high-grade glioma) | X |  |  |
| 13 | Soft tissue/bone sarcoma | X | X | X |
| 17 | Soft tissue/bone sarcoma | X | X | X |
| 18 | Soft tissue/bone sarcoma | X | X | X |
| 19 | CNS (anaplastic ependymoma) | X | X |  |
| 20 | CNS (pilomyxoid astrocytoma) | X | X |  |
| 21 | CNS (diffuse midline glioma) |  | X | X |

ATRT, atypical teratoid rhabdoid tumour; CNS, central nervous system; GI, gastrointestinal; NA, not available.

^a^ Patient had no measurable disease at baseline.

**Supplementary Table 5.** Baseline characteristics and outcomes in patients with CNS tumours.

| **Assigned patient number** | **Tumour type** | **Site of primary tumour** | **Prior anticancer regimens, n** | | **Prior RTx (yes/no)** | **Best overall response with avelumab (duration of SD at cutoff)** | **Vital status at last follow-up** | **Best response to prior therapy** |
| --- | --- | --- | --- | --- | --- | --- | --- | --- |
| 9 | Atypical teratoid/rhabdoid tumour | Cerebellopontine angle | 2 | | Yes | PD | Dead | CR with vincristine + methotrexate + etoposide + cyclophosphamide + cisplatin + tamoxifen + isotretinoin with autologous stem cell transplant + RTx; unknown response with metronomic chemotherapy including isotretinoin + celecoxib + etoposide + cyclophosphamide + temozolomide |
| 11 | Glioblastoma | Intracranial | 2 | | Yes | PD | Dead | Unknown response with temozolomide and temozolomide maintenance |
| 12 | High-grade glioma; WHO grade IV | Pineal gland | 2 | Yes | | PD | Dead | Unknown response with bevacizumab;  unknown response with temozolomide + lomustine |
| 14 | Pilocytic astrocytoma; *BRAF* V600E mutation positive | Brain, NOS | 2 | No | | SD (30.3 months) | Alive | PD with carboplatin + vincristine;  unknown response with thioguanine + procarbazine + lomustine + vincristine |
| 15 | Astrocytoma of spinal cord (WHO grade II); *BRAF* V600E mutation negative | Spinal cord | 1 | No | | SD (24.7 months) | Alive | PD with carboplatin + vincristine |
| 19 | Anaplastic ependymoma | Ventricle, NOS | 2 | Yes | | PD | Dead | PD with vincristine + cyclophosphamide + etoposide + cisplatin maintenance;  PD with fluorouracil |
| 20 | Pilomyxoid astrocytoma; *BRAF* V600E mutation negative | Suprasellar | 2 | No | | SD (12.7 months) | Alive | SD with carboplatin + vincristine induction;  PR with carboplatin + vincristine maintenance |
| 21 | Diffuse midline glioma (primary site, thalamus); WHO grade IV; H3 K27M mutation | Thalamus | 4 | Yes | | SD (2.4 months) | Dead | Non-CR/non-PD with temozolomide; PD with temozolomide + lomustine; PD with procarbazine + lomustine;  PD with cisplatin + vincristine + etoposide + cyclophosphamide + carboplatin + ifosfamide |

Assigned patient numbers are taken from the overall patient population (see Supplementary Table 3).

CNS, central nervous system; CR, complete response; NOS, not otherwise specified; PD, progressive disease; PR, partial response; RTx, radiotherapy; SD, stable disease; WHO, World Health Organization.

**Supplementary Table 6.** PD-L1 status in all patients.

| **Assigned patient number** | **Tumour type** | **PD-L1 status (any cutoff)** | **PD-L1 tumor cell staining at any intensity, %** | **Reason for NE or details of staining intensity** |
| --- | --- | --- | --- | --- |
| 1 | Colon cancer, sigmoid | Not evaluable | Not evaluable | Tissue washed off primary antibody slide |
| 2 | Desmoplastic small round cell tumour | Negative | <1% | – |
| 3 | Osteogenic sarcoma | Not evaluable | Not evaluable | No tumor seen in sample |
| 4 | Rhabdomyosarcoma | Not evaluable | Not evaluable | No tumor seen in sample |
| 5 | Rhabdomyosarcoma | Negative | <1% | – |
| 6 | Rhabdomyosarcoma | Negative | <1% | – |
| 7 | *BCOR-CCNB3* sarcoma | Positive | 25% | 25% staining at 2+ intensity |
| 8 | Osteosarcoma | Negative | <1% | – |
| 9 | Atypical teratoid/rhabdoid tumour | Negative | <1% | – |
| 10 | Undifferentiated sarcoma | Positive | 5% | 5% staining at 2+ intensity |
| 11 | Glioblastoma | Negative | <1% | – |
| 12 | High-grade glioma; WHO grade IV | Not evaluable | Not evaluable | Inadequate sample |
| 13 | Ewing sarcoma | Not evaluable | Not evaluable | Unacceptable sample |
| 14 | Pilocytic astrocytoma; *BRAF* V600E mutation positive | Positive | 95% | 25% staining at 2+ intensity, 70% staining at 3+ intensity |
| 15 | Astrocytoma of spinal cord (WHO grade II); *BRAF* V600E mutation negative | Positive | 80% | 20% staining at 1+ intensity, 30% staining at 2+ intensity, 30% staining at 3+ intensity |
| 16 | Rhabdomyosarcoma | Not evaluable | Not evaluable | No viable invasive tumor cells in the sample |
| 17 | Malignant peripheral nerve sheath tumour/ schwannoma/acoustic neuroma | Positive | 2% | – |
| 18 | Rhabdomyosarcoma | Negative | <1% | – |
| 19 | Anaplastic ependymoma | Negative | <1% | – |
| 20 | Pilomyxoid astrocytoma; *BRAF* V600E mutation negative | Negative | <1% | – |
| 21 | Diffuse midline glioma (primary site, thalamus); WHO grade IV; H3 K27M mutation | Negative | <1% | – |

NOS, not otherwise specified; WHO, World Health Organization.


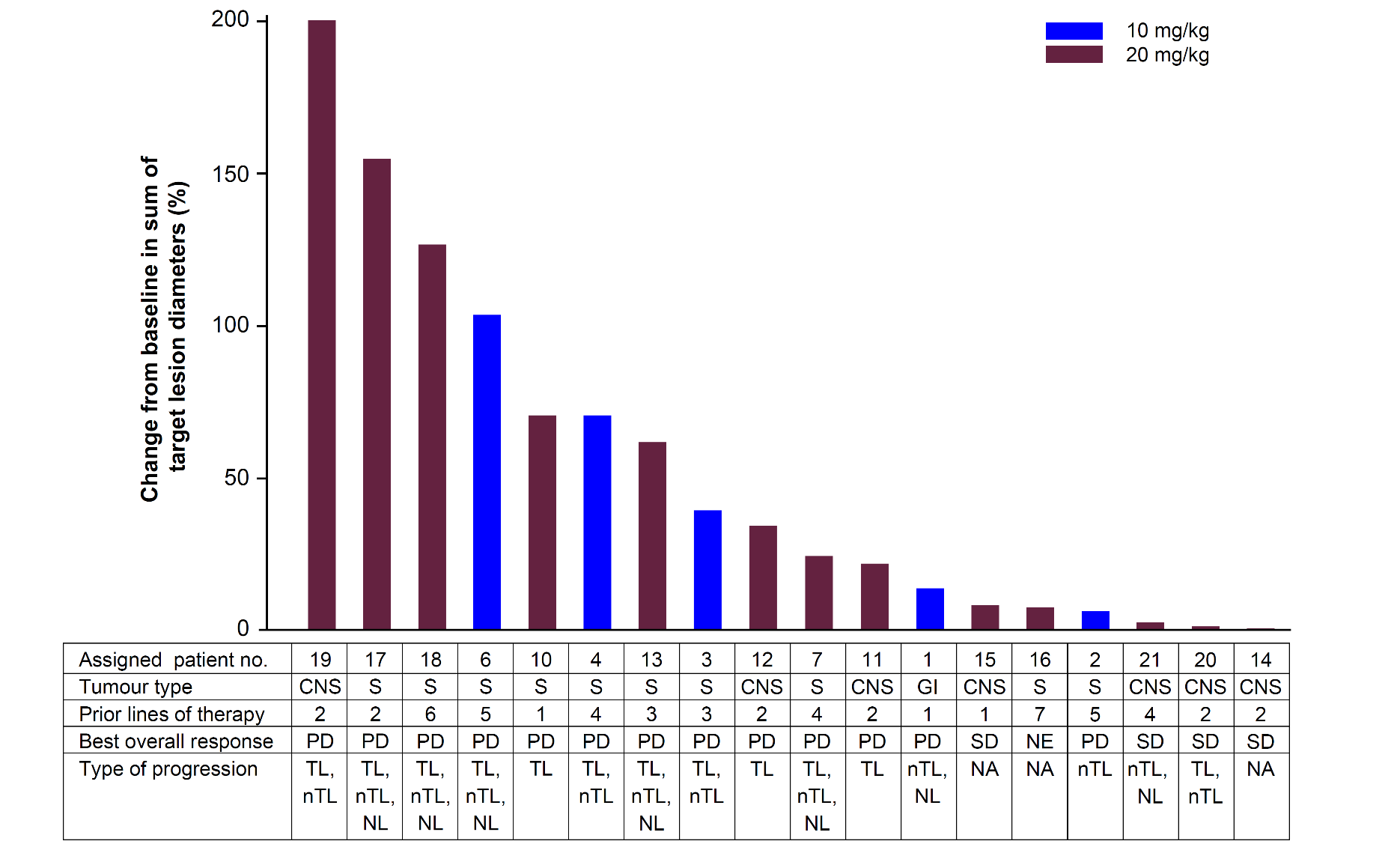
**Supplementary Fig. 1** Best change in target lesions from baseline in evaluable patients (baseline and postbaseline data available; n=18). Increases greater than 200% are shown as 200%. CNS, central nervous system; GI, gastrointestinal; NA, not available; NE, not evaluable; NL, new lesion; nTL, nontarget lesion; PD, progressive disease; S, sarcoma; SD, stable disease; TL, target lesion

**Supplementary Fig. 2** (A) Duration of SD from enrolment (n=4) and (B) change in target lesions over time vs baseline in three patients with low-grade central nervous system tumours who had SD for >12 months with avelumab.

^a^The patient with astrocytoma of the spinal cord was classified as having ongoing SD with avelumab because the defined criteria for PD per RECIST 1.1 were not met (tumour did not increase by ≥5 mm).

PD, progressive disease; SD, stable disease

**A**


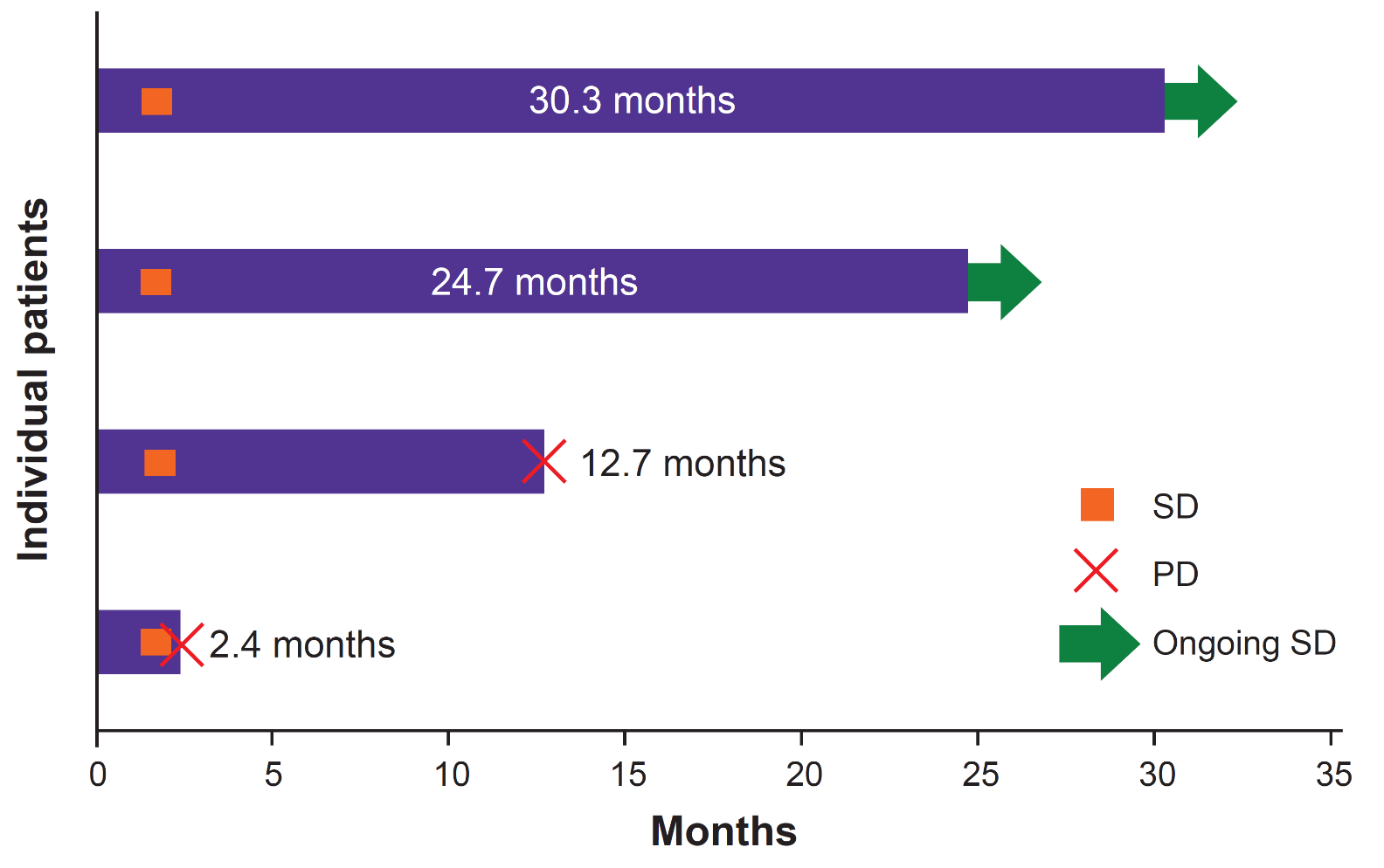


**B**


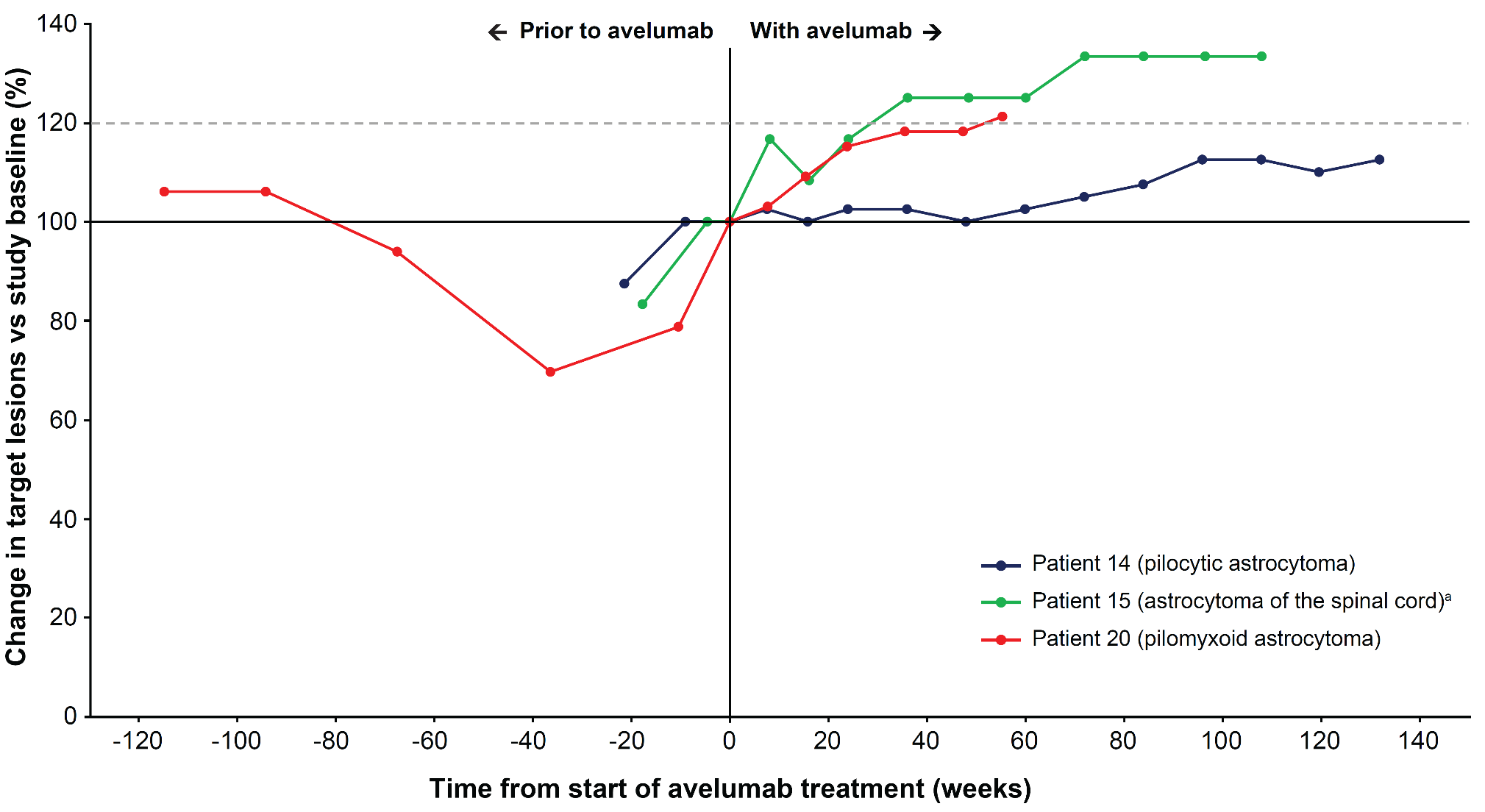


**Supplementary Fig. 3** Progression-free survival (A) and overall survival (B) in avelumab dose cohorts

**A

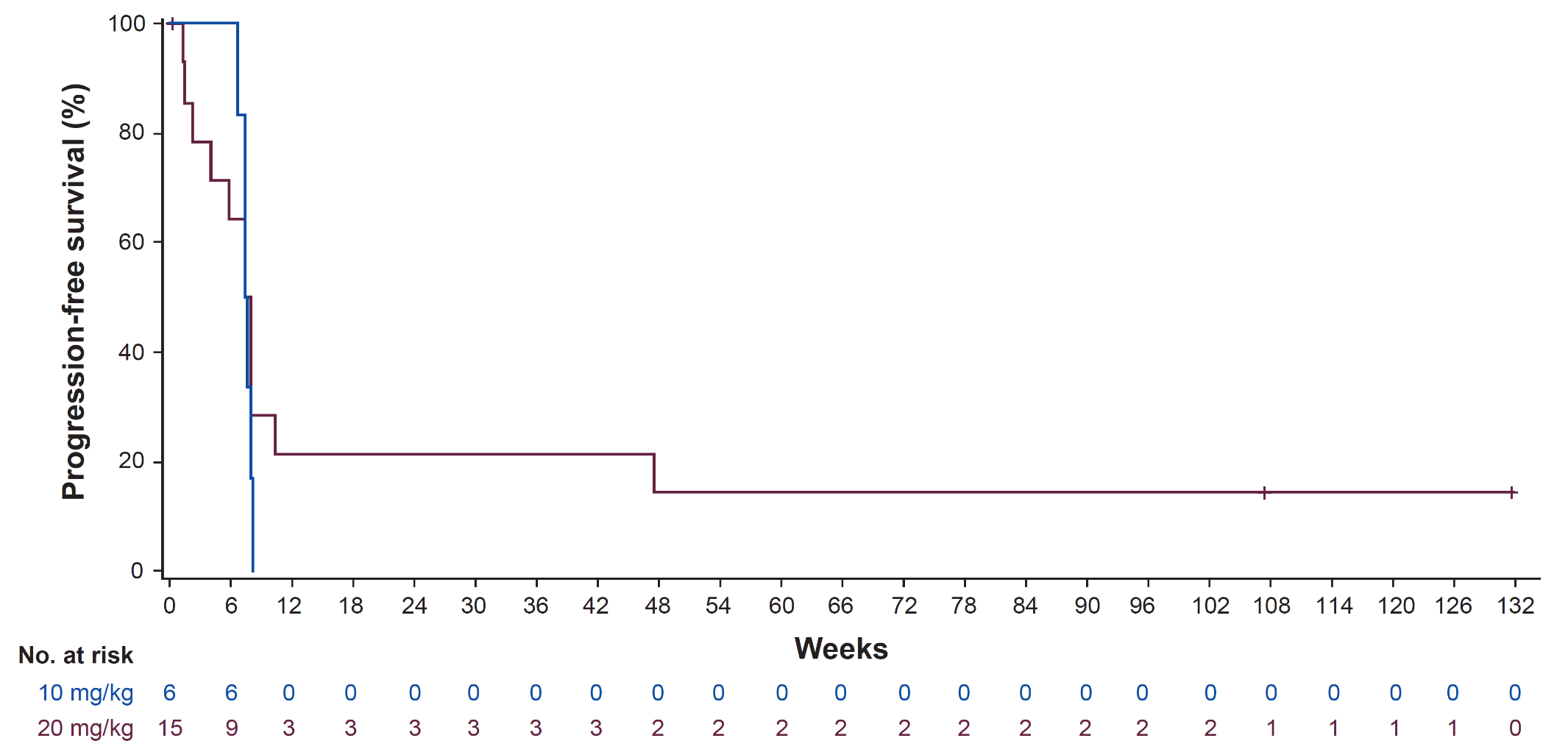
**

**B**

**
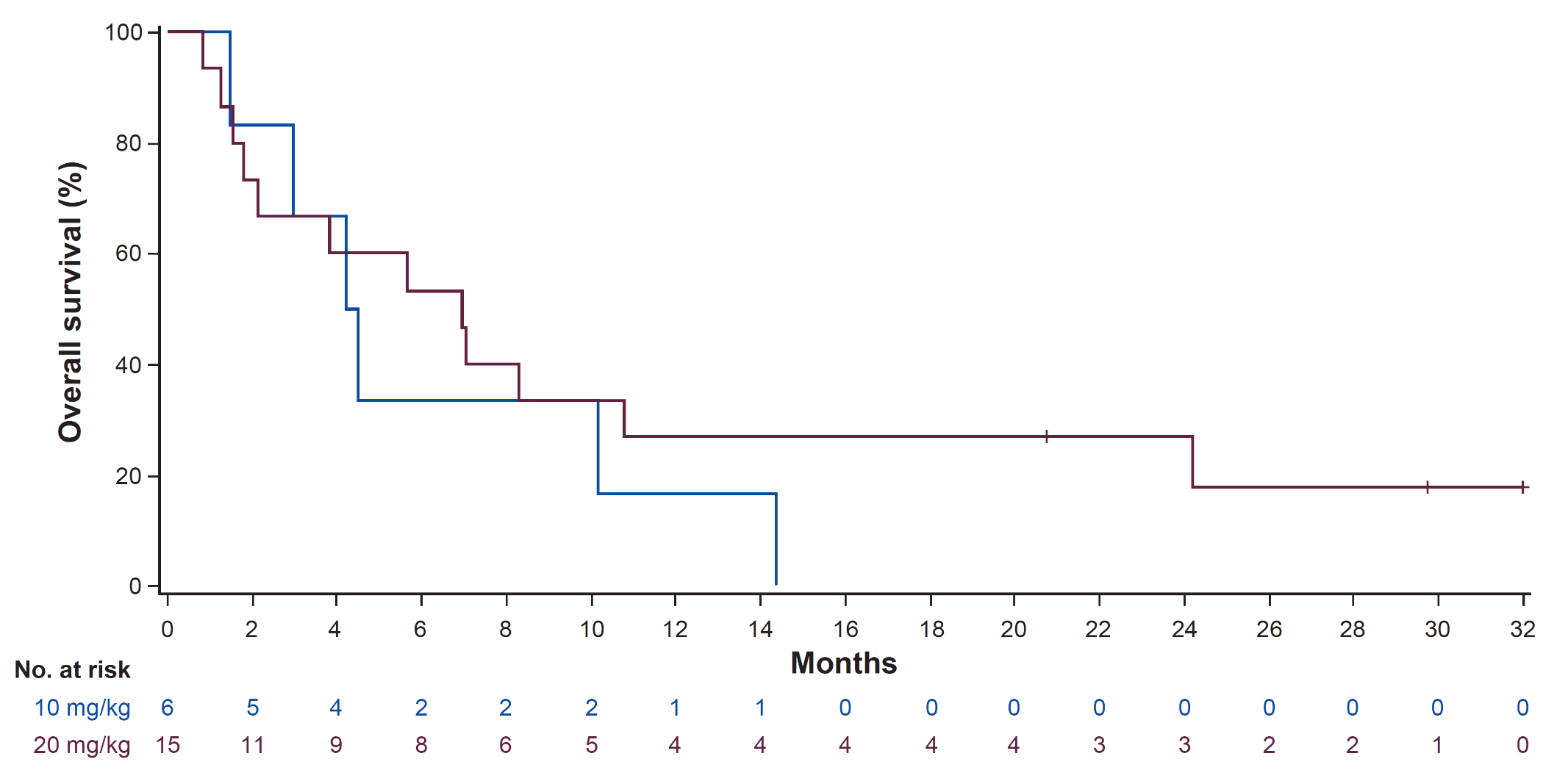
**
